# The power of music: impact on EEG signals

**DOI:** 10.1007/s00426-024-02060-6

**Published:** 2025-01-06

**Authors:** Basma Bahgat El Sayed, Mye Ali Basheer, Marwa Safwat Shalaby, Hala Rashad El Habashy, Saly Hasan Elkholy

**Affiliations:** Department of Neurology and Clinical Neurophysiology Unit, Faculty of Medicine-Cairo University, Cairo, Egypt

## Abstract

**Introduction:**

Music is known to impact attentional state without conscious awareness. Listening to music encourages the brain to secrete neurotransmitters improving cognition and emotion.

**Aim of work:**

Analysis of QEEG band width while listening to two music types, identifying different cortical areas activated and which genre has a similar effect to relaxed EEG.

**Methods:**

This is a cross-section interventional analytic study including 76 normal subjects, 55 of them are females (72.37%). Participants listened to 10 min of a single audio track during EEG recording, consisting of (1 min of silence, 3 min of Egyptian folk music, 3 min of silence, then 3 min of Egyptian instrumental classic music (without any lyrics). We analyzed QEEG bands at each brain region during different tracks. The power ratio index (PRI) was calculated for each region, and then the interhemispheric difference was compared.

**Results:**

The participants’ ages ranged from 15 to 26 with a mean 16.73 ± 2.37 years. PRI showed a significant increase in the frontal and occipital regions during listening to folk music compared to the silent epoch, where p < 0.001 and p = 0.023, respectively. In the frontal and temporal regions, the classic music epoch evoked the highest PRI interhemispheric difference compared to the folk music epoch, where p = 0.004 and p < 0.001, respectively.

**Conclusion:**

Egyptian folk music has significantly slowed the brain rhythm, particularly in the frontal region, compared to classic music, supporting the hypothesis of a momentary reduction of cognitive capacities by the noise. Classic music was evidently associated with a relaxed state EEG.

## Introduction

Listening to music can encourage the brain to secrete neural substrates, which improve cognition and emotion (Ashby & Isen, 1999). The improvement of neuroendocrine, cardiovascular, and digestive system functions and stimulating the cerebral cortex increase the release of acetylcholine, which improves cognition (Bleibel et al., 2023).

Music helps patients sustain long-term sensory input, enhance the ascending reticular activating system, and increase levels of cortical neuronal activity, increasing arousal (Gerber, 2005). It impacts the limbic system directly and can cause the cerebral cortex to develop excitation foci (Sun & Chen, 2015).

Quantitative electroencephalogram (QEEG) develops from EEG quantification by mathematical computer technology. It converts amplitude changes with time into a digital signal and presents perceptual, measurable parameters (Sun & Chen, 2015).

One of the powerful tools that offers profound and objective comprehensions of music impact on the brain is spectral analysis of brain waves. Further investigation of this area may increase the comprehension of how the brain handles music and can offer added insights into the use of music in therapeutic settings (Kucikiene & Praninskiene, 2018; Rahman et al., 2020).

This study aims to analyze QEEG band relative power while listening to two different music types, identify the different cortical areas that got activated, and determine which type resulted in EEG signals resembling relaxed EEG.

## Methods

This cross-sectional interventional analytic study included 76 subjects of both genders. Subjects with a history of hearing complaints, chronic illnesses, epilepsy, and/or receiving psychiatric treatment were excluded.

All participants underwent a routine EEG recording using EBNeuro Galileo NT (PMS USA), model Mizar.B8351037899. (version 3.61). We performed QEEG analysis using WinEEG software (3.1).

A headphone was used to conduct 10 min of a single audio track all through the recording (supplementary data is available). The audio track started simultaneously with the EEG recording and consisted of 1 min of silence, 3 min of Egyptian folk music, 3 min of silence, and 3 min of Egyptian classic music. The participants were informed that they would hear different sounds during the examination, with no interaction expected from them. They received instructions to close their eyes throughout the audio playback.

All subjects filled out a questionnaire (in Arabic) that was designed by the authors to identify any ailments that would affect the outcome of the study. (Appendix 1). It includes inquiries about handedness, hearing problems, chronic illnesses, epileptic or psychiatric history, pre-examination music practice, and preference.

A single EEG interpreter inspected the EEG visually for artifacts or any abnormal classification. Three epochs are chosen for quantitative analysis: first between the 2nd and 3rd, then between the 5th and 6th, and then between the 8th and 9th minutes of recording.

Data was analyzed using Jamovi; 2.3.28 (Love et al., 2024) (Sydney, Australia). The power ratio index (PRI) was calculated by dividing the relative power sum of slow bands (delta and theta) by fast bands (alpha and beta). The interhemispheric difference in PRI was obtained by subtracting left hemisphere values from corresponding right hemisphere values.

Categorical data was expressed in terms of count and percent (%) (Table 1). The normality of the distribution of numerical data was assessed using the Shaprio–Wilk test for normality. Normally distributed numerical data is expressed in terms of mean and standard deviation (SD) and compared using the Student T-test or ANOVA. Not normally distributed numerical data is expressed in terms of median, 25th, and 75th percentiles. Comparisons between two independent groups were carried out using the Mann–Whiteny U test, and comparisons between two independent groups were carried out using one-way ANOVA (Kruskal–Wallis test), followed by post hoc analysis using Dwass–Steel–Critchlow–Flinger pairwise comparisons. Paired comparisons of non-parametric data were done using repeated measures ANOVA (Friedman test), followed by pairwise post hoc analysis using Durbin-Conover. A repeated measures ANOVA was used for comparisons between subgroups after ensuring the homogeneity of the data using Levene’s test (Hollander et al., 2015).

**Table 1:** Descriptive data

Variable	Percent (count)
Gender	75.3% (55) females
Handedness	92.1% (70) right-handed
Play a musical instrument	2.6% (2) yes
Music preference (in general)	15.8% (12) don’t like music at all
	25% (19) prefer folk music
	59.2% (45) prefer classic music
Listen to music during study/work	86.8% (66) don’t listen to music
Music preferred during EEG recording	71.1% (54) classic music

## Results

The participants’ ages ranged from 15 to 26 years, with a mean of 16.73 ± 2.37 years; 55 of them were females (72.37%). All of them are/were attending public high schools with an average sociocultural level.

The PRI showed a significant increase in the frontal and occipital regions during the folk music epoch compared to the silent epoch, where p < 0.001 and p = 0.023, respectively. And in the frontal region only when compared to the classic music epoch, p < 0.001. Moreover, the total sum of PRI (from all brain regions) also showed a significant increase during the folk music epoch compared to the silent epoch, where p = 0.02 (Table 2).

**Table 2:** PRI in different brain regions during the three epochs

Region	Folk music epoch			Silent epoch			Classic music epoch			P1	P2	P3
	25th	50th	75th	25th	50th	75th	25th	50th	75th			
Frontal	11.03	18.1	23.77	9.79	13.9	19.71	9.01	12.9	18.74	< 0.001	< 0.001	0.37
Central	5.05	7.76	14.14	4.39	7.61	12.11	3.70	6.97	12.27	0.06	0.12	0.74
Parietal	1.8	4.12	6.96	1.78	3.43	6.02	1.66	3.34	6.93	0.19	0.37	0.69
Temporal	3.97	7.44	12.80	3.65	5.98	10.27	2.89	5.99	11.64	0.05	0.22	0.46
Occipital	2.98	4.58	8.36	2.17	4.47	8.68	2.23	4.29	7.47	0.02	0.17	0.37
Total PRI	3.88	6.46	10.58	3.40	5.73	9.40	2.99	5.38	9.45	0.02	0.09	0.46

*PRI* Power ratio index, *P1* folk vs silent, *P2* folk vs classic, *P3* classic vs silent

On comparing PRI at corresponding regions in between hemispheres, there was a significant difference between the classic music epoch and the folk music epoch, where the classic music evoked the highest interhemispheric PRI difference in the frontal region compared to the folk music epoch (with the left hemisphere recording higher values than the right hemisphere), where p = 0.004.

While, in the temporal region, classic music also elicited the highest interhemispheric PRI difference, the left hemispheric values were much lower than the right hemispheric values, and this difference was also significant compared to the folk music epoch, where p < 0.001.

The classic music epoch interhemispheric PRI difference was closer in values as well as behavior (where the left hemisphere readings were lower than the right hemisphere reading) to the silent epoch readings except at the temporal region, where it was significantly higher during the classic music epoch, p < 0.001.

Consistently, during the classic music epoch and the silent epoch, the right hemisphere recorded higher values in almost all brain regions. However, this relationship was reversed in the folk music epoch.

It is worth mentioning that with the classic music, the left frontal region showed the highest PRI differences, while the left temporal region showed the lowest differences (Table 3).

**Table 3:** Interhemispheric PRI difference (Left–Right)

Region	Folk music epoch		Silent epoch		Classic music epoch		P1	P2	P3
	Mean	Median	Mean	Median	Mean	Median			
Frontal	0.61	– 0.12	1.78	0.66	2.82	2.29	0.07	0.004	0.28
Central	– 2.2	– 0.85	– 1.82	– 0.53	– 1.43	– 0.3	0.26	0.29	0.94
Parietal	– 0.14	0	– 0.96	– 0.17	– 0.44	0.09	0.33	0.99	0.33
Temporal	0.13	0.46	– 0.29	0.17	– 3.87	– 2.21	0.58	< 0.001	< 0.001
Occipital	0.16	0.04	0.41	0.09	0.75	0.05	0.87	0.42	0.52

*PRI* power ratio index; *P1* folk vs. silent; *P2* folk vs. classic; *P3* classic vs. silent

## Discussion

Functional magnetic resonance imaging (fMRI) and positron emission tomography (PET) studies have proven that listening to music activates many brain areas, as different brain regions process various properties of musical appreciation. There are several connections between the prefrontal cortex, superior temporal gyrus, and parietal lobe's precuneus (Hughes & Fino, 2000; Menon & D'Esposito, 2022).

Evaluating brain activity using electroencephalography (EEG), magnetoencephalography (MEG), or functional magnetic resonance imaging (fMRI) can be used to assess its responses to distinct temporal resolutions of music. The sampling for EEG and MEG is on the scale of 1–10 ms, and the sampling for fMRI is on the scale of 0.72–3 s. (Garza Villarreal et al., 2011; Vuust et al., 2022).

Music activates a series of cognitive and affective components of the brain, which are neural-specific matrixes (Peretz & Zatorre, 2005).

Guo et al. (2020) discussed the neural mechanisms of sad music in alleviating pain. This may help recognize the effects of music on the modulation of pain, which presents a possible usefulness in clinical settings.

The past two decades of research into the effect of music on the brain have created a foundational understanding of how the brain processes music. As part of this journey, there are many unresolved questions. Recent experiments have supplemented the current understanding of music and the brain, which primarily relies on studies of western music and participants (Vuust et al., 2022). This study, however, utilized local music and participants to achieve its objectives.

Our studied subjects, 15.8%, did not like music at all. Mas-Herrero et al. (2014) elaborated on a slightly higher percentage. Only 2.6% of our study group used to play music. Throughout the world, it’s estimated that 13% of adults between the ages of 16 and 24 play a musical instrument; however, this percentage depends on several factors, including age, socioeconomic status, and geographic location (Hincliff, 2021).

In the current study, about 59.2% preferred classic music in general, and 71.1% preferred the classic music played while the EEG examination was performed. According to Goltz and Sadakata (2021), about 20% of people included in their study prefer non-vocal (i.e., instrumental), calm, and classical music in the background. The variation in musical preferences among people with very similar cultural backgrounds is likely due to repeated listening to musical pieces, and possibly it expresses group affiliation (Corrigall & Schellenberg, 2015; Madison & Schiolde, 2017).

The assumption that background music elevates the cognitive abilities of the students is preferred to the theory that listening to music while engrossed in complex cognitive tasks leads to impairment of performance (Avila et al., 2011). A small portion (13.2%) of our studied sample mentioned they would like to listen to music while studying or working. This percentage is much lower than a study conducted in India in 2016 on a similar age group that showed 60% listen to music while studying. Moreover, the Indian study showed that the type of music preferred while studying was classical music in 43% of their sample studied. They postulated reasons to listen to music while studying as follows: music helps them to pay attention while studying (47%), keeps their mind calm (29%), would prevent sleepiness (17%), and would block any external interference (7%). Gonzalez and Aiello (Gonzalez & Aiello, 2019) conducted a study that suggests background music can prevent attention distraction and improve task performance (Kumar et al., 2016).

Some studies claim that music distracts concentration, interferes with mood, and that performance is worse when music is on, just not liking it, the personal habit not to have music on, and not finding a space where music is on. In this study sample, the major contribution of socioeconomic factors is suspect (Goltz & Sadakata, 2021).

The EEG power ratio index (PRI), which reflects the relative power of the slow waves (delta and theta; δ + θ) in relation to the fast waves (alpha and beta; α + β) (δ + θ/α + β value), is one of the most sensitive indices in brain function research (Sun & Chen, 2015).

The current study showed a significant increase in PRI in the frontal and occipital regions during the folk music epoch compared to silence. This relationship was also reflected in the total PRI values. However, compared to the classic music epoch, the folk music epoch showed a significant increase in PRI in the frontal region only.

Some previous studies mentioned that pop music serves as a distractor in cognitive performance; pop and hip-hop tend to reduce performance more than instrumental classical music (Avila et al., 2011) . Additionally, the rhythm and volume of the music can modify the outcome, as loud and fast music causes the greatest impairment (Perham & Currie, 2014; Thompson et al., 2012).

Kumar et al. (2016) assessed the type of music that works best in improving the concentration of participants; 75% of their study group seemed to have better concentration with slow music. A facilitating effect on reading comprehension has been demonstrated with classical background music. Additionally, studies have shown that classical background music enhances reading efficiency and speed (Kallinen, 2002; Li et al., 2012).

As the music became noisier, students' attention started to decline. This advocates that either they prefer to listen to calming or quiet music while working on a task to recuperate attentiveness, or that as the music grows noisier and speedier, it eventually is perceived as pure noise (Kumar et al., 2016).

Consequently, according to the relations between theta, preference, and cognitive state, regional slowing in the prefrontal cortex (PFC) is intensely correlated to both upbeat emotion and cognitive state (Aftanas & Golocheikine, 2001).

We found that the classical music epoch revealed a significant reduction of slow waves recorded from the left in relation to the right temporal regions when compared to the silence epoch. According to Nakamura et al. (1999), by comparing the periods of silence and music, listening to music recruited new areas of the brain into the active processes. This effect reveals the effect of music on cognitive processes, as music-evoked memory recall. This recruitment was more elaborated during the Ding study (Ding et al., 2019). They performed electrocorticography recordings obtained from 10 epilepsy patients of both genders implanted with subdural electrodes while subjects were listening to familiar instrumental music. Listening to music evoked a gamma band in the supramarginal, precentral, and inferior frontal gyri during the early phase up to 500 ms, then a delta band was evident during the following phase. Researchers attributed this to the relaxing effect of classic music (Ding et al., 2019).

Rauscher et al. (1993) made a claim that, after listening to Mozart's sonata for two pianos (K448) for 10 min, normal subjects showed significantly better spatial reasoning skills than after periods of listening to relaxation instructions designed to lower blood pressure or silence. Hence, the term "Mozart's effect" was coined. It also enhanced the synchrony of the firing pattern of the right frontal and left temporoparietal areas of the brain, which persisted for 12 min. This was associated with an enhanced beta band in both temporal and right frontal regions (Rideout & Laubach, 1996; Sarnthein et al., 1997).

When listening to music (even not Mozart), greater beta power results, particularly in the precuneus bilaterally. Hughes and Fin argued in 2000 that any "Mozart effect" is due to the enjoyment arousal occasioned by this music and would not take place in the absence of its appreciation (Hughes & Fino, 2000; Nakamura et al., 1999).

Almost all brain areas studied were impacted by listening to music, but mainly frontal, temporal, and occipital to a lesser extent. Egyptian folk music has significantly slowed the brain rhythm in almost all brain areas, particularly the frontal one, in relation to classic music, supporting the hypothesis of a momentary reduction of cognitive capacities by the noise effect. Finally, relaxing EEG was more found with classic music.

## Data Availability

No datasets were generated or analysed during the current study.
